# Our roles are not at ease: The work of engaging a youth advisory council in a mental health services delivery organization

**DOI:** 10.1111/hex.13302

**Published:** 2021-07-09

**Authors:** Eugenia Canas, Nadine Wathen, Helene Berman, Paula Reaume‐Zimmer, Srividya N. Iyer

**Affiliations:** ^1^ Faculty of Information & Media Studies Western University London ON Canada; ^2^ Centre for Research and Education on Violence Against Women and Children Western University London ON Canada; ^3^ Arthur Labatt Family School of Nursing Western University London ON Canada; ^4^ Centre for Research on Health Equity and Social Inclusion Western University London ON Canada; ^5^ Mental Health and Addiction Services Bluewater Health and Canadian Mental Health Association Lambton Kent ON Canada; ^6^ ACCESS Open Minds (Pan‐Canadian Youth Mental Health Services Research Network) Douglas Research Centre Montréal QC Canada; ^7^ Department of Psychiatry McGill University Montréal QC Canada; ^8^ Douglas Mental Health University Institute Montréal QC Canada

**Keywords:** mental health services, patient‐oriented research, service transformation, service user involvement, youth engagement, youth mental health

## Abstract

**Objectives:**

There is growing policy impetus for including youth voices in health services research and health system reform. This article examines the perspectives of professionals in a mental healthcare organization charged with engaging young people as advisors in service transformation.

**Methods:**

An institutional ethnography of a youth mental health services organization in Ontario, Canada, was conducted. Fieldwork consisted of twelve months of observation of meetings, interviews with youth advisors and adult service providers, with subsequent text analysis of engagement training and policy materials. The present article reports data from six adult professionals and related field observations.

**Results:**

Service providers’ efforts to engage youth were observed in three areas: a) supporting youth's development as advisors, b) retaining and deepening youth participation while waiting for organizational change and c) embedding relationships between youth and adults at various levels within the system of care. This work denotes existing tensions between the values and ideals of youth engagement and the everyday demands of services delivery.

**Conclusion:**

In this setting, a fundamental dimension of this work consisted of negotiating tensions between the policy enthusiasm for engagement and its realization in a health services context. In describing these contextual challenges, we outline implications for consideration by other youth mental health services. Engagement efforts that are authentic and sustained require resources and flexibility, and leadership commitment to instil service users’ perspectives throughout multiple levels within the organization.

## 1 INTRODUCTION

Policy and research agendas focused on youth mental health services increasingly argue for the inclusion of young people as advisors to mental health services reform.^1^, ^2^, ^3^ The role of youth advisors has gained prominence in complex service transformation projects.^4^, ^5^ However, youth engagement has not been critically explored through the perspectives of service providers and organizational administrators. Doing so will help us to better understand how this policy impetus translates into individual practice and organizational operations. In this article, we share findings from an institutional ethnography^6^ of a mental health services delivery organization. Particular attention will be paid to the points of tension and opportunity between the expectations of youth engagement mandates and their realization.

## 2 BACKGROUND

The last two decades have seen various levels of commitment towards youth mental health systems improvement. In Canada, scholars have lamented the system's limited provision of access to timely and appropriate care, and its lack of youth and family engagement.^3^, ^7^, ^8^ The salient trend in service reform is towards integrated youth services, which bring together a range of services for young people and their families, integrating mental health and primary care with vocational and other social services under a commitment to holistic and client‐centred care.^9^ Several organizations represent this model of care. In Canada, these include ACCESS Open Minds, Foundry, Youth Wellness Hubs Ontario, Kickstand and Projet Aire ouverte. International examples are headspace in Australia and Israel, Jigsaw in Ireland, @ease in Netherlands and allcove in the United States. This orientation requires contextual attention: approaches to integrated youth services may differ and be informed by different actors in every setting. To that end, the meaningful engagement of youth and families, alongside perspectives from diverse populations within each community, has been deemed essential to the implementation of integrated youth services.^4^, ^9^ Youth’s input in service design and evaluation is now seen as a requirement if care is to be developmentally informed, contextually relevant and engaging.^10^ This impetus for advisory roles for youth is now a requirement in strategies for systems change, and a criterion for funding success in service reform and research initiatives.^7^, ^9^, ^11^

### 2.1 Engaging young people as advisors

Shaw^12^ ^(p. 16)^ defines meaningful youth engagement as “the intentional establishment and support for the involvement of young people in the design, creation, coordination, implementation and evaluation of the processes, practices and decisions that shape civic life." Arguments for engagement have been put forward in the areas of positive youth development,^13^ participatory research^14^ and patient involvement.^15^, ^16^ The instrumental role of patient and service user involvement is described as a means to generate knowledge regarding 1) health inequities, namely remediable differences in health status relating to class, culture, gender, sexuality and ethnicity; and 2) differences in access to health‐care provision and health outcomes according to such differences.^17^ As a policy shift, youth engagement aligns with the principle of participation, under the rights‐based approach for health equity articulated in the World Health Organization's Commission for the Social Determinants of Health (2008) and adopted in the 2030 Agenda for Sustainable Development Goals.^1^, ^18^

Research examining engagement has included projects with young adults with psychiatric disabilities^19^, ^20^ and with youth who are trained as research assistants.^14^, ^21^ Evaluation of youth engagement initiatives has focused on individual outcomes for youth, such as increases in capacity, self‐efficacy and professional skills.^15^, ^22^ A second area of impact relates collective benefits to the community: knowledge generated from participatory approaches is considered to be more culturally relevant and connected to people's lived experiences, thus benefiting research, policy and programming activities.^23^, ^24^

## 3 CONTEXT FOR STUDY

ACCESS Open Minds, a Canadian multidisciplinary research network focused on youth mental health system transformation, is an acronym that stands for Adolescent/young adult Connections to Community‐driven, Early, Strengths‐based and Stigma‐free services. The project has involved a five‐year mental health system process of organizational and network change in 14 communities in six provinces and one territory.^3^, ^7^

ACCESS Open Minds is supported by the Canadian Institutes of Health Research (CIHR) Strategy for Patient Oriented Research (SPOR) funded through a partnership between CIHR and a philanthropic foundation. SPOR refers to a continuum of research that engages patients as partners, focusing on patient‐identified priorities with the goal of improving patient outcomes.^25^ Since inception, ACCESS Open Minds has included the significant input of youth and family stakeholder groups, from envisioning the project and its core values, to the implementation and evaluation of service transformation at each site. Project governance includes national youth and family/caregiver councils that function in partnership with working groups in the project. Family and youth councils also exist at many of the 14 sites. Engagement is both a core value and one of the service transformation objectives of ACCESS Open Minds. This dual conceptualization guides the implementation of engagement activities and creates accountability for particular outcomes and experiences resulting from youth engagement.

This study examined the engagement of a youth advisory council (YAC) to understand the processes by which their perspectives informed the organizational culture, plans and operations, and the contextual care challenges of a service delivery organization in Chatham‐Kent, Southwestern Ontario. This organization offers free services as part of the public health system in the province, serving a small urban centre and its surrounding rural areas (population 108,000), including two First Nations communities. The service site launched publicly as a part of the larger ACCESS Open Minds initiative in May 2016.^26^ During the fieldwork for this study, this site, ACCESS Open Minds Chatham‐Kent, underwent its initial design and planning phases for a walk‐in, one‐stop storefront service dedicated to youth mental health. A YAC was formed to provide input into this stage of the site's development, as well as its ongoing function in the future. At the time of this study, the youth advisors had been recruited through radio advertising, and the community and social networks of youth and service providers. They met as a council in person approximately once a month and participated in other working groups and committees within the organization. They received honoraria for their time and expertise.

## 4 METHODOLOGY

Institutional ethnography (IE) is a qualitative method informed by the field of sociology.^6^ Components of institutional ethnography share epistemological and methodological features with other types of ethnographies,^27^ with "modern" ethnography as a method having been primarily developed in the field of anthropology.^28^ IE aims to describe how certain forms of knowing, such as official discourses or policy narratives about a certain issue, can authoritatively replace, undermine and also uphold other forms of knowing, such as people's understandings of their daily life and work practices. Analysis in IE focuses in detail on the actualities of people's daily experiences to describe work processes and to understand how these are shaped by institutional structures, discourses or practices.^29^ IE scholars have previously described how workers in mental health services enact processes of empowerment of persons with lived experience of mental illness.^30^ They have also examined how changes happen in the direct service work of mental health services organizations,^31^ and how young people are not getting their needs met in their interactions with public institutions.^32^

### 4.1 Methods

This article is one part of a larger IE study that explores how youth's knowledge is taken up to inform mental health services and that involved interviews with youth advisors and adult service providers, as well as observations of meetings and text analysis.^33^ This approach to data adhered to the IE commitment to collecting careful observations of everyday life by working with diverse sources and small informant samples.^29^, ^30^ The current article focuses on the work of service providers. It is informed by five meeting observations, seven interviews and four follow‐up discussions with six service provider persons employed by the organization as site administrators, research and clinical coordinators, and youth counsellors, social workers and/or peer navigators. Informant selection was purposeful to represent a range of experiences within the adult professional group that interacted with youth advisors; two of the adults interviewed had been hired (0.5 full‐time equivalent) to support the YAC.

IE conceptualizes participants as expert informants, relying on their accounts as the starting point for inquiry, then driving analysis upwards towards a macro‐understanding of trans‐local activities, discourses and values—termed within IE as "ruling relations".^6^ Semi‐structured interviews were one hour in length; they were recorded and transcribed. Analysis was inductive and iterative: it began during the interview process and was guided by an IE focus on people's descriptions of their experiences while seeking their connection to organizational structures and practices.^29^ Canas (first author) recorded and transcribed the interviews and recorded the meeting observations through field notes and research memos. In keeping with Mykhalovskiy and McCoy,^34^ specific terms served as anchors through data gathering and analysis; a measure exemplified by the term "engagement work" as an orienting concept that directed attention towards the wide range of practices of the service providers in relation to the youth. Data were read multiple times to distil salient groupings related to the research questions and to index clues to the connection between the individual experience and ruling relations. This process of indexing was guided by the analytic approach of Bisaillon and Rankin (2012).^27^ The evolving index of connections was discussed with the co‐authors over a period of six months, leading up to the articulation of findings and discussion.

This study received approval from the Research Ethics Board at the first author's university and at the hospital host to ACCESS Open Minds Chatham‐Kent where the research was conducted. All names used are pseudonyms.

## 5 FINDINGS

Findings in this article describe the work of the service providers with the youth advisors, in the context of the professional culture and policies of the organization (ACCESS Open Minds Chatham‐Kent). Forms of work identified through this study follow.

### 5.1 Work supporting youth's development as advisors

Leading to the site launch, and proceeding from it, this organization held continuous consultation with family and youth groups, resulting in the creation of an initial YAC composed of 12 youth. The service providers used various techniques to ensure youth would feel comfortable and welcome in the physical space of the site and during meetings. Particular skillsets, such as relational training, flexibility and the capacity for self‐reflect, were noted as useful in working with the youth advisors.

The service providers prepared youth ahead of meeting or activity in the organization, describing how agendas would unfold and who would be there. Pam, who coordinated the YAC, noted this was important, "so they know what to expect." She assumed that these meetings would be new to youth and that they would need support to feel comfortable and understand how things work in the organization.

At the same time, the service providers placed great importance on the difference between showing the youth how the organization works and telling the youth how to think. Lynn, a peer counsellor, spoke of how she described her work to the youth advisors during a brainstorming session to design clinical spaces in the future site. "From my view, I can think of what happens, so I described that to them," she said. “What does our desk need to look like? What are some safety concerns? You wouldn't know unless you actually worked here…. But it's still having both sides and coming together."

To support youth advisors in bringing their own views to the organization, the service providers facilitated multiple opportunities where youth advisors could develop their communication skills and messaging. The service providers also translated the organizational language—and the language of the mental health sector—into understandable terms, consistently spelling out acronyms and providing background information whenever new youth advisors joined the group.

As observed, the adults’ support sometimes went explicitly against the professional pace of the organization. For example, in a meeting of the Facilities Planning Group, Pam and Rose slowed down the discussion to set up a question that engaged the views of a youth advisor. Once the youth shared their opinions, Pam asked a probing question to encourage further description, and Rose commented on the youth advisor’s insights, inviting others to engage with the points made. In doing this, both adults countered a fast‐paced meeting agenda to create conditions where a young person could articulate their ideas. By engaging others in the discussion, they worked to embed the youth advisor’s perspective amidst the thinking of others, including hospital administrators and community stakeholders. It is notable that Rose was chairing this meeting, because she had the authority to set the pace of the discussion.

This intentional slowing down went against the pace of working culture at the site, as was noted by other service providers. Betty saw this shift as necessary, saying, "It takes time for youth to figure out what they want." Some service providers saw the professional culture of health services itself as a challenge to meaningful engagement. Fran described this pressure in her direct service work with young people, saying, "You have to shut off the professional realities side, in order to genuinely listen to youth."

This supportive and sometimes repetitive approach to engagement created tension for some service providers; it brought into question their professionalism. Several meetings with youth focused on trust‐ and rapport‐building. Consequently, they did not have reportable outcomes beyond youth having a positive experience. In those cases, the service providers found themselves offering minimal updates to management boards. Pam said, "As an adult professional, it is uncomfortable to feel like there is nothing new to report. It looks like no work is happening because there is nothing reportable." Others felt how time spent with the youth advisors did not align with organizational notions of what was plannable or codifiable as work. Describing her coordination of youth advisory activities, Maria said, "Our roles are not at ease. It involves becoming comfortable with things developing organically." She added, "Constant change can look like failure from a professional‐performance side."

This tension was negotiated by the service providers in the ways they tracked their work with, and on behalf of, youth with others in the organization and community. Pam said, "We need to redefine what success looks like—what [the YAC members] give to the project and what the project gives them." Maria asked, "How to measure success as a council? Is it dropout numbers, skills development? How we observe their participation changing?" They felt that evaluation of engagement, and of the involvement of service users in general, required a departure from the conventional work in the organization.

### 5.2 Waiting work

While meetings at the site tended to move too fast for youth's comfort, the envisioned transformation of services moved slowly. Processes that took time included ethics reviews, the hiring of new positions, changes in management and lease negotiations for the service site. Throughout these waiting periods, youth tended to disengage or move on to other phases of their lives, such as post‐secondary education in other communities. The service providers continually recruited new youth advisors, restarting processes of building trust and familiarizing them to the organizational language and culture in order to keep up council membership numbers stable. The service providers also created new opportunities for long‐standing youth advisors to stay engaged, even remotely.

Administrators in the organization supported all of ‘waiting work’ by allocating staff time and resources and by promoting these events to community stakeholders and other local project partners. For service providers, having an active and visible YAC during this waiting period played a critical role in ensuring the sustainability of the service site. Rose said, "If we'd even just started up, I think it would've been easy to say, ‘This isn't a priority.’ But we'd already established a presence, we had so much community buy‐in, I think that it demonstrated the value."

### 5.3 Working to embed trusting relationships between youth and the system of care

The values of collaboration and authenticity, core characteristics of ACCESS Open Minds, were noted repeatedly as important guides in the work with youth advisors. Maria expressed an ongoing concern with "being able to deliver on these values" not only during the moments of interacting with the youth advisors, but also in how she reflected on her work with them. She added, "Sometimes you have to revise, undo, re‐invent how things work because you see that the trajectory it took you on is not authentic engagement."

Service providers at other levels of the organization showed this commitment to working authentically by focusing on transparency. The realities of what was possible for the organization sometimes contrasted the youth advosors' enthusiasm for change, or the ambition of their ideas during brainstorming sessions. Lynn said, "I think it's just important to be transparent, and honest and open about things." The service providers spent significant time explaining timelines, budgets and the nature of service provision in the community. Updates on ongoing projects and full discussions of how decision making unfolded were key agenda items in all youth council meetings. For example, during the sessions that focused on choosing a physical space for the site, the YAC members heard for the first time how choosing a certain space over another would have repercussions on how many full‐time positions could be hired to serve youth in the area. All participants felt that time spent creating transparency supported stronger relationships between youth and the organization, because it helped youth understand the role of cutbacks and risk aversion in decision making. Lynn noted that a failure to support youth in understanding "how things work at the site" can harm existing trust, because youth feel their ideas are not being heard. Rose noted that, when youth advisors are not informed of the realities of service delivery, their perspectives risk being discounted or perceived as unreasonable by other stakeholders. She said, "[b]ringing youth in and co‐creating and working side by side doesn't mean you hand everything over to them. We're responsible for making sure that we guide them, and do the coaching that helps them. …I think that's an important piece of why the youth need to be on every single working group and council."

Rose's comment underscores a key observation throughout fieldwork: namely that the commitment to include youth advisors occurred at various levels in the organization and brought youth into relationship with diverse groups. Youth advisors' involvement shaped the presence of this site in the community at large. In addition to participation in working groups, youth were significantly engaged as speakers and facilitators in community‐wide events, such as the site launch. They also contributed to hiring and evaluation processes, providing input on job descriptions and interviewing panels for research and direct service positions. Some of the service providers interviewed had been hired as a result of youth advisors' input, which represented a subtle but significant shift on how youth advisors were perceived in relation to the organization and its evolution.

By being open to change their scope of practice and organizational processes as a direct result of the youth advisors' presence, these service providers shifted engagement from a series of interpersonal exchanges between themselves and the youth advisors, to a relationship between youth and the system of care.

## 6 DISCUSSION

In keeping with an institutional ethnographic approach, we framed youth advisory engagement as a form of work—that is, any activities that take intention, effort and time and that people do in particular places, under definite conditions and with definite resources.^6^ At a fundamental level, this study underscores that establishing and sustaining the authentic engagement of a YAC requires time and effort on the part of adults at all levels of the organization. That this service site recognizes engagement as work is evidenced in the hiring, training, time allocation and reporting structure established for individuals who support the YAC and perform various visible and invisible engagement tasks as part of their professional remit. The support of site administrators was a salient feature at ACCESS Open Minds Chatham‐Kent. Adults in various roles worked with youth by fostering communication across the organization, and providing mentorship, new skills and relationship building. Such leadership support has been deemed both crucial and rare in other youth engagement initiatives in mental health services and research.^9^, ^24^, ^35^ The ACCESS Open Minds approach to youth engagement as both a principle and an objective of the national initiative may be providing an additional support, increasing both the will to engage youth, and the organizational structure and accountability to ensure that engagement takes place meaningfully.

This study's detailed examination of what the service providers do to keep youth advisors meaningfully engaged revealed three types of work: a) supporting youth's development as advisors, b) retaining and deepening youth's engagement while waiting for organizational change and c) embedding relationships between youth and adults at various levels within the system of care. That these activities require effort, rather than occurring fluidly and naturally in the context of the organization, suggests a dissonance between what youth bring and need as advisors, and the way a mental health services site operates. This finding corroborates Nichols’ (2014) observation that mainstream institutions fail to adequately serve young people as a result of misalignments between what youth need, and the system's prioritization of risk, efficiency and accountability concerns.^32^

The work of engaging youth advisors at this site unfolded inseparably from, and sometimes at odds with, what mattered at the organization. In their activities with youth, the service providers had to respond to ebbs and flows in service demand, changes in administration, the pace of hospital and research processes, and uncertainties in the landscape of funding and government support. As such, what worked for youth advisor engagement was both intensive and unique to its time and place, suggesting that although the overall principles for engagement may be similar across ACCESS Open Minds sites, their particular implementation will vary according to the organizational context and population of youth engaged. The highly contextual nature of this work has been noted in other youth mental health services settings^20^, ^35^ and in service user involvement in general.^16^

Consistent with the effortful nature of youth advisor engagement, tensions were visible. The first tension was related to the professional expectations of what it means to interact with youth, at what pace and resulting in what sort of reportable outputs. This work—of making youth feel comfortable enough to speak up, supporting them to understand the mental health system, or explaining previous decision making in the organization—has not been previously codified as work for service providers and administrators. It is knowledge and relational work that may not always fit with the institutional culture of healthcare employees. The service providers sometimes went against what was expected of them at meetings, specifically the fast pace, professional jargon and acronyms that characterize healthcare culture and which have been noted to have exclusionary effects.^15^, ^19^ In doing this despite the unease they felt, these adults enacted the youth engagement values of inclusion and knowledge democratization.^14^ This process has been acknowledged by scholars and youth engagement practitioners to take time.^12^, ^20^, ^24^

A second tension identified was related to youth's enthusiasm for change, evidenced in the scope of their ideas and brainstorming sessions, and the institutionalized risk aversion of health services organizations. The service providers spent considerable effort providing youth with contextual information of what was possible at the service site at that time, outlining the implications of choices. Their approach was designed to make youth's input "meaningful" in the context of the organization, and their efforts towards transparency and honesty in communication underscored the commitment to authentic, rather than the tokenistic or symbolic involvement of youth.^14^, ^15^ However, the risk remains that youth advisors, through repeated exposure to what is possible within health services research and delivery, are over time acculturated to think exactly like the organization, thus negating the original intent of engaging them. This risk has not been explored in the area of youth engagement.

A final, broader tension relates to the misalignment between some of the ideals of youth engagement (e.g., the fervour for things to be youth‐led) and their realization in practice in a service delivery setting. In response to this tension, service providers who had the organizational power to do so embedded youth advisors in working groups and committees where they could contribute to decision making, and where their perspectives could be considered alongside others in the organization. Service providers in positions of leadership saw themselves as responsible for mentoring youth advisors and exposed them to experiences across the organization and with community partners so that youth would function as partners and collaborators, rather than merely users of the mental health system. This approach aligns with fundamental principles of engagement and youth‐adult partnerships, including coaching, dialoguing and connecting young people to institutional resources and community leaders.^8^, ^12^, ^22^ In the process of embedding youth advisor roles into the service site working groups, the service providers supported the establishment of a more complex relationship between young people and the system of care.

## 7 STUDY LIMITATIONS

This study described youth engagement activities and experiences at one site, embedded in (and resourced by) a larger project with a specific youth engagement goal. The YAC, at the time of this study, did not include young people of colour, or from any of the First Nations communities in the area. As such, the work of engagement on the part of the service providers may need to be expanded to include strategies that may be more effective at engaging youth from visible minority and Indigenous groups. This study is not representative of other contexts, either within or outside of mental health services. However, the IE approach allowed in‐depth assessment of the service providers’ activities, in various data sets across time. Studies on different models of youth engagement, or of advisory councils in different health services contexts, should be an urgent goal of future research.

## 8 IMPLICATIONS FOR POLICY OR PRACTICE

While this article focused on a framing of youth engagement as work for adults, what youth advisors do also merits examination as a form of work. Understanding the nature of youth's efforts and the forms of compensation and satisfaction that youth advisors find in these roles may contribute to more diverse and equity‐oriented engagement. Calls for institutional support for engagement—in the form of remuneration for youth involved and the employment of youth‐engaging adults with specialized skills and experience in the area—have appeared in discussions of participatory research^14^ and mental health services organizations.^20^, ^21^, ^35^ This study addresses these calls with a detailed view of how professionals in this setting, when tasked with engagement, employ their time and expertise, as well as the kinds of organizational supports needed to enact the values of engagement. In doing so, it adds to the emerging body of literature that enhances how the practical, everyday work undertaken by teams of frontline staff make their experiences, and those of the populations they serve, better understood mutually.^23^

Hiring decisions, and support for these service providers to build emergence and flexibility into their work with youth, was a result of leadership commitment and resourcing towards youth engagement at both the site level and from the national guidance of the ACCESS Open Minds project. The framing of youth engagement as both a high‐level value and an objective served as a protective framework to values‐oriented work in context. It increased resources devoted to engagement efforts, gave legitimacy to ways of operating that were new or appeared to counter professional culture and expanded possibilities of how young people can interact with a mental health services organization.

While other organizations may be guided by different funding and health system contexts and may not receive explicit support for service user involvement from their funding bodies, we note the general trend towards such engagement as an integral component of building contextually relevant services and care.^10^, ^17^ Other models of engagement exist, and an advisory council may not suit every service organization. We suggest organizations consider their existing expertise and time resources to find out what matters to the populations they serve. Resources may also take the form of training support from other organizations poised to advance engagement efforts.

Informed by our work, we have outlined high‐level recommendations for other health services settings seeking to engage youth in the Table below.

Table 1: Recommendations for youth advisory engagement in health services settings

**Table:** 

Protect staff time within the organization to create welcoming spaces and opportunities for youth advisors and to transfer updates and perspectives from the organization to youth and from youth to the organization.
Seek opportunities for youth advisors to interact with and belong to working groups and decision‐making tables at various levels of the organization.
Reflect on the values and objectives of engaging youth as advisors with persons in positions of leadership in the organization. Ensure that youth advisors have opportunities to build relationships and exchange views with organizational administrators.
Consider the values of youth or service user engagement when making hiring and staffing decisions in an organization.
Create policies around remuneration and non‐financial means of compensating youth for their time and expertise. These should be discussed with youth, along with other terms of engagement.

## 9 CONCLUSION

In this study, we identified the nature of the work required to bring youth as advisors to various levels of organizational decision‐making. Institutional ethnography served to detail forms of work that had not previously been coded as such, and yet supported the development and inclusion of youth perspectives. In light of the macro‐level policy impetus for youth engagement, it becomes timely to underscore that it is resource‐intensive, with needs and implications defined by its context. Health services organizations undertaking the engagement of youth and other populations as advisors to the development of services may benefit from this clarity about its realities.

## AUTHORS’ CONTRIBUTIONS

This article is based on Canas’ dissertation research which was completed under the supervision of Nadine Wathen and Helene Berman, and the advisement of Srividya Iyer. Eugenia Canas’ role in this article included conceptualization, methodology, data acquisition and curation, formal analysis and writing—original draft. Nadine Wathen contributed to conceptualization and writing—review and editing. Helene Berman contributed to writing—review and editing. Paula Reaume‐Zimmer is a community collaborator and contributed to writing—review and editing. Srividya N. Iyer contributed to conceptualization, supervision and writing—review and editing.

## Data Availability

Data available on request due to privacy/ethical restrictions.

## References

[1] Patel V, Saxena S, Lund C, et al. The Lancet Commission on global mental health and sustainable development. The Lancet. 2018;392(10157):1553‐1598. 10.1016/S0140-6736(18)31612-X 30314863

[2] Kieling C, Baker‐Henningham H, Belfer M, et al. Child and adolescent mental health worldwide: evidence for action. The Lancet. 2011;9801:1515‐1525. 10.1016/S0140-6736(11)60827-1 22008427

[3] Malla A, Shah J, Iyer S, et al. Youth mental health should be a top priority for health care in Canada. Can J Psychiatry. 2018;63(4):216‐222. 29528719 10.1177/0706743718758968 PMC5894919

[4] McGorry P, Bates T, Birchwood M. Designing youth mental health services for the 21st century: examples from Australia, Ireland and the UK. Br J Psychiatry. 2013;202(54):30‐35. 10.1192/bjp.bp.112.119214 23288499

[5] Settipani CA, Hawke LD, Cleverley K, et al. Key attributes of integrated community‐based youth service hubs for mental health: a scoping review. Int J Ment Health Syst. 2019;13(1):52. 31367230 10.1186/s13033-019-0306-7 PMC6651922

[6] Smith DE. Institutional ethnography: A sociology for people. Rowman Altamira; 2005 Jun 3.

[7] Iyer SN, Boksa P, Lal S, et al. Transforming youth mental health: a Canadian perspective. Ir J Psychol Med. 2015;32(1):51‐60. 31715701 10.1017/ipm.2014.89

[8] Henderson JL, Chaim G, Brownlie EB. Collaborating with community‐based services to promote evidence‐based practice: Process description of a national initiative to improve services for youth with mental health and substance use problems. Psychol Serv. 2017;14(3):361. 28805421 10.1037/ser0000145

[9] Halsall T, Manion I, Iyer SN, Mathias S, Purcell R, Henderson J. Trends in mental health system transformation: integrating youth services within the Canadian context. Healthc Manage Forum. 2019;32(2):51‐55. 30799661 10.1177/0840470418808815

[10] Pullmann MD, Ague S, Johnson T, et al. Defining engagement in adolescent substance abuse treatment. Am J Community Psychol. 2013;52(3–4):347‐358. 24046184 10.1007/s10464-013-9600-8

[11] Malla A, Iyer S, McGorry P, et al. From early intervention in psychosis to youth mental health reform: a review of the evolution and transformation of mental health services for young people. Soc Psychiatry Psychiatr Epidemiol. 2016;51(3):319‐326. 26687237 10.1007/s00127-015-1165-4

[12] Shaw K. Practice in perspective: Youth engagement in the Canadian context. Unpublished master’s thesis (2012), University of Victoria. Victoria, British Columbia, Canada. Quoted by: Shaw‐Raudoy K, McGregor C. Co‐learning in youth‐adult emancipatory partnerships: the way forward?. *Int J Child Youth Family Stud* 2013 Aug 21;4(3.1):391‐408.

[13] Lerner JV, Phelps E, Forman YE, et al. Positive youth development. In: Lerner RM, Steinberg L, eds. Handbook of Adolescent Psychology. Individual Bases of Adolescent Development, 3rd edn. Hoboken, NJ: Wiley; 2009:524‐558.

[14] Berman H, Richardson/Kinewesquao C, Elliott K, Canas E eds. Everyday Violence in the Lives of Youth. Nova Scotia: Fernwood Publishing; 2020.

[15] Monson K, Thurley M. Consumer participation in a youth mental health service. Early Interv Psychiatry. 2011;5(4):381‐388. 22032552 10.1111/j.1751-7893.2011.00309.x

[16] Greenhalgh T, Hinton L, Finlay T, et al. Frameworks for supporting patient and public involvement in research: systematic review and co‐design pilot. Health Expect. 2019;22(4):785‐801. 31012259 10.1111/hex.12888 PMC6737756

[17] Beresford P. User involvement, research and health inequalities: developing new directions. Health Soc Care Community. 2007;15(4):306‐312. 17578391 10.1111/j.1365-2524.2007.00688.x

[18] World Health Organization . Closing the Gap in A Generation. Geneva: World Health Organization; 2008.

[19] Delman J. Participatory action research and young adults with psychiatric disabilities. Psychiatr Rehabil J. 2012;35(3):231. 22246121 10.2975/35.3.2012.231.234

[20] Elliott NB, Canas E, Paquette A, et al. Youth Engagement and Community‐Based Participatory Research: The Bipolar Youth Action Project. Thousand Oaks, CA: SAGE Publications Ltd; 2017.

[21] Lincoln AK, Borg R, Delman J. Developing a community‐based participatory research model to engage transition age youth using mental health service in research. Fam Community Health. 2015;38(1):87. 25423247 10.1097/FCH.0000000000000054 PMC4256677

[22] Minkler M, Wallerstein N, eds. Community‐Based Participatory Research for Health: From Process to Outcomes. San Francisco, CA: John Wiley & Sons; 2011.

[23] Soleimanpour S, Brindis C, Geierstanger S, et al. Incorporating youth‐led community participatory research into school health center programs and policies. Public Health Rep. 2008;123(6):709‐716. 19711652 10.1177/003335490812300607 PMC2556716

[24] Woodgate RL, Zurba M, Tennent P. Advancing patient engagement: youth and family participation in health research communities of practice. Res Involv Engagem. 2018;1:9. 10.1186/s40900-018-0094-2 PMC5846254 29560275

[25] Canadian Institutes of Health Research . Strategy for patient‐oriented research–patient engagement framework.

[26] Reaume‐Zimmer P, Chandrasena R, Malla A, Joober R, Boksa P, Shah JL. Transforming youth mental health care in a semi‐urban and rural region of Canada: a service description of ACCESS open minds Chatham‐Kent. Early Interv Psychiatry. 2019;13(Suppl 1):48‐55. 31243909 10.1111/eip.12818 PMC6771628

[27] Bisaillon L, Rankin J. Navigating the politics of fieldwork using institutional ethnography: strategies for practice. Forum Qual Soc Res. 2012;14:1‐26.

[28] Clair RP. The changing story of ethnography. Expressions of Ethnography: Novel Approaches to Qualitative Methods. Albany, NY: State University of New York; 2003:3‐26.

[29] DeVault ML, McCoy L. Institutional ethnography: using interviews to investigate ruling. Critical Strategies for Social Research. Lanham, Maryland: Rowman & Littlefield Publishers; 2004:191.

[30] Townsend E. Institutional ethnography: a method for showing how the context shapes practice. Occup Ther J Res. 1996;16(3):179‐199.

[31] Griffith AI, Smith DE, eds. Under New Public Management: Institutional Ethnographies of Changing Front‐Line Work. Toronto, Canada: University of Toronto Press; 2014.

[32] Nichols N. Youth Work: An Institutional Ethnography of Youth Homelessness. Toronto, Canada: University of Toronto Press; 2014.

[33] Canas E. Working with Youth as Stakeholders in Mental Health System Transformation: An Institutional Ethnography of a Service Organization in ACCESS Open Minds. Dissertation. Western University; 2019. Accessed August 20, 2020. https://ir.lib.uwo.ca/etd/6327/

[34] Mykhalovskiy E, McCoy L. Troubling ruling discourses of health: Using institutional ethnography in community‐based research. Crit Public Health. 2002;12(1):17‐37.

[35] Heffernan OS, Herzog TM, Schiralli JE, et al. Implementation of a youth‐adult partnership model in youth mental health systems research: Challenges and successes. Health Expect. 2017;20(6):1183‐1188. 28295940 10.1111/hex.12554 PMC5689223

